# Dual Targeted Therapy With Risankizumab and Upadacitinib in Refractory Ulcerative Colitis

**DOI:** 10.14309/crj.0000000000002173

**Published:** 2026-06-04

**Authors:** Padmavathi Srivoleti, Nireet Dhillon, Leo Boneschansker

**Affiliations:** 1 Division of Gastroenterology, Department of Internal Medicine UMass Chan Medical School, Worcester, MA; 2 Department of Internal Medicine, UMass Chan Medical School, Worcester, MA

**Keywords:** ulcerative colitis, IBD, upadacitinib, risankizumab

## Abstract

Medically refractory acute severe ulcerative colitis (UC) remains a major therapeutic challenge. We describe a 39-year-old woman with multiple prior treatment failures who presented with a steroid-refractory UC flare. Given that she was symptomatic despite high-dose upadacitinib, dual targeted therapy with upadacitinib and risankizumab was initiated. The patient subsequently achieved rapid and sustained clinical remission with complete endoscopic and histologic mucosal healing. No serious adverse events were observed during 10 months of follow-up. This case highlights the potential role of dual targeted therapy with upadacitinib and risankizumab as a treatment strategy for selected patients with refractory severe UC.

## INTRODUCTION

Ulcerative colitis (UC) is a chronic inflammatory bowel disease (IBD) characterized by mucosal inflammation limited to the colon. Management of moderate-to-severe UC has been transformed by the development of advanced biologics and targeted small molecules. Despite these effective monotherapies, a subset of patients exhibit refractory disease that fails to achieve adequate response, and novel approaches are needed to improve treatment outcomes. Case series and retrospective cohort data suggest potential benefit of combining Janus kinase inhibitors with anti-interleukin (IL) 23 agents in Crohn's disease, but evidence in UC remains limited.^1,2^ We report a case of successful management of severe, medically refractory UC using dual targeted therapy with upadacitinib and risankizumab.

## CASE REPORT

A 39-year-old woman with a 13-year history of left-sided UC (Montreal E2 S3) presented with worsening abdominal pain and bloody diarrhea (10–15 bowel movements per day). She previously lost response to mesalamine, adalimumab, vedolizumab, and more recently to a reduced dose of tofacitinib. On admission, inflammatory markers were elevated, with a C-reactive protein (CRP) of 90.4 mg/L, fecal calprotectin of 2,740 μg/g, and white blood cell count of 14 × 10^9^/L, consistent with an acute UC flare (Truelove/Witts Index-Severe). She was treated with intravenous methylprednisolone; however, she remained symptomatic with CRP trending up to 273 mg/L. Given persistent symptoms despite intravenous steroids and prior loss of response to anti-tumor necrosis factor, decision was made to transition to upadacitinib.

The patient reported prior passive exposure to tobacco and denied any personal or family history of diabetes mellitus, hypertension, coronary artery disease, or venous thromboembolism. She has a first-degree relative (mother) with UC. Her body mass index was 25.5. She denied current use of oral contraceptives but had taken them 6 months before admission. She was neither pregnant nor planning for a pregnancy.

Before initiating upadacitinib, viral hepatitis screening was performed. Hepatitis B testing (total core antibody and HBsAg) was negative, whereas hepatitis B surface antibodies were positive, consistent with immunity. Hepatitis C serologies were also negative. She received the Shingrix vaccine after starting upadacitinib. Baseline laboratory studies, including a complete blood count, comprehensive metabolic panel, and lipid profile, were obtained and monitored throughout treatment. Standard inpatient venous thromboembolism prophylaxis was provided.

She had rapid biochemical improvement with upadacitinib 45 mg once daily (CRP 46 mg/L). However, 4 days after upadacitinib induction, while being transitioned to per os (by mouth) prednisone 40 mg, she experienced recurrence of symptoms and rising CRP (120 mg/L) Figure 2. The colorectal surgery team evaluated the need for proctocolectomy but determined that immediate surgical intervention was not necessary. A flexible sigmoidoscopy was performed, which demonstrated severely inflamed mucosa characterized by ulceration in the rectosigmoid colon, consistent with severe UC (Mayo sub score 3). Histology showed chronic active colitis and cytomegalovirus immune-staining was negative. Intravenous Solu-Medrol 30 mg twice daily was resumed, and the upadacitinib dose was increased to 30 mg twice daily. This escalation is not standard practice for UC and was extrapolated from data on tofacitinib. The tofacitinib in acute severe ulcerative colitis trial demonstrated that induction with high-dose tofacitinib in patients with acute severe UC reduced the need for medical rescue therapy or colectomy.^3^ The patient received upadacitinib 30 mg twice daily for 10 days, during which she demonstrated clinical and biochemical improvement, including normalization of CRP (<3 mg/L). She did not require infectious prophylaxis during this period but remained hospitalized under close monitoring for infections and thromboembolic events. She was subsequently discharged on upadacitinib 45 mg daily and oral prednisone 40 mg, with a taper of 10 mg every 7 days. On this regimen, she achieved a brief period of clinical remission.

**Figure 1.**
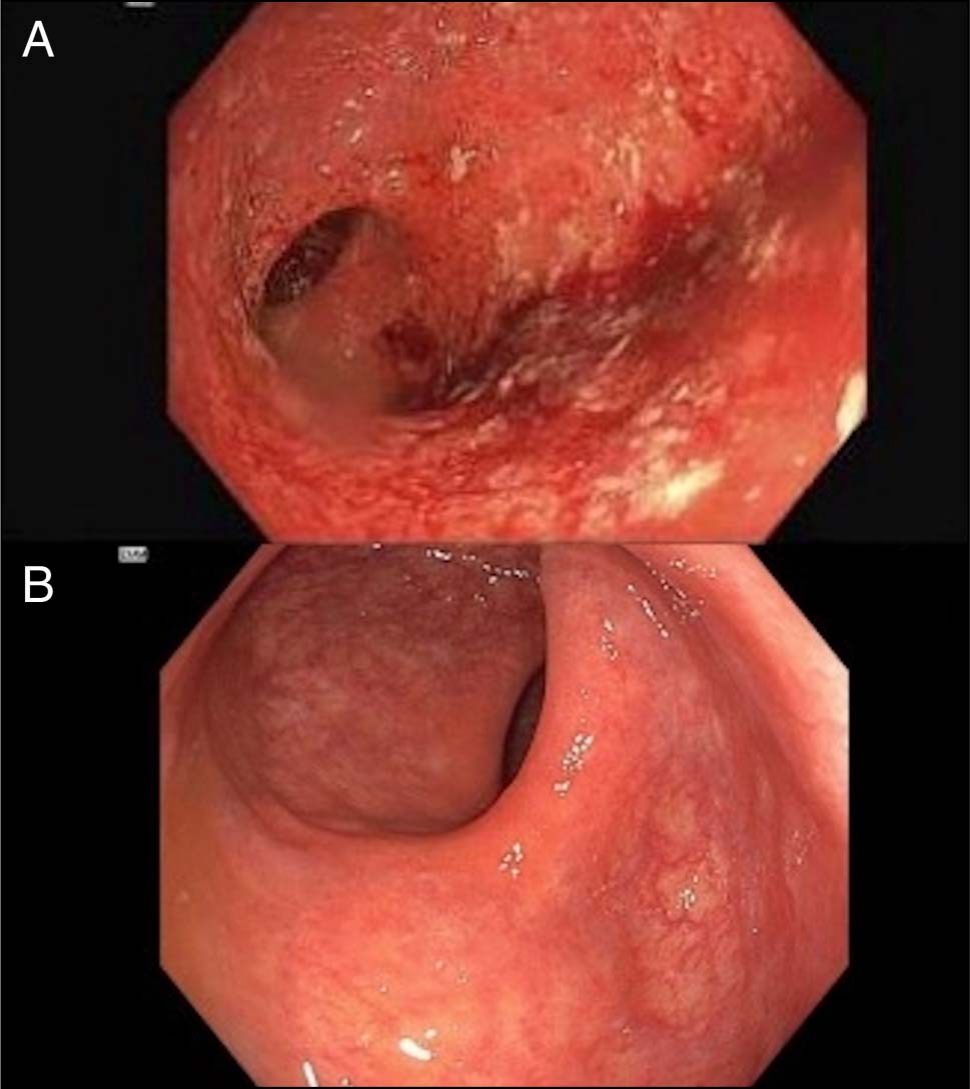
Endoscopic evaluation of colonic mucosa before (A) and after (B) upadacitinib and risankizumab dual targeted therapy.

**Figure 2.**
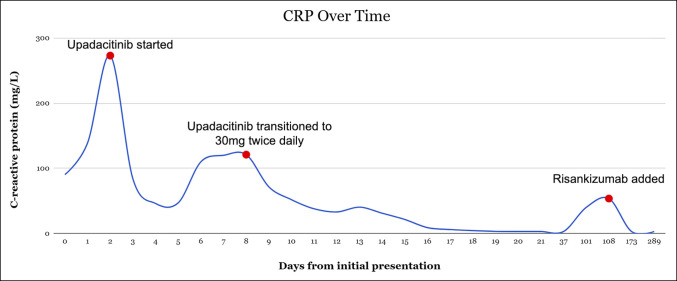
C-reactive protein trend throughout the initiation of dual targeted therapy with upadacitinib and risankizumab.

Two months after her discharge, after completion of the prednisone taper, she developed a recurrent flare with 7 bloody bowel movements daily and an elevated CRP (40 mg/L) and fecal calprotectin (4,360 μg/g) (Figure 2). Given ongoing inflammation despite upadacitinib therapy, decision was made to pursue dual targeted therapy, and risankizumab was initiated as adjunctive therapy. After receiving 3 induction doses, her inflammatory markers normalized (CRP <3 mg/L; fecal calprotectin 6 μg/g). Colonoscopy performed 5 months after initiation of dual targeted therapy, showed endoscopic remission with histology demonstrating normal colonic mucosa with no granulomas or dysplasia (Mayo subscore 0) (Figure 1). She remains in clinical remission on 30 mg of upadacitinib and risankizumab with stable biomarkers. Serial lipid profile monitoring demonstrated mild hypercholesterolemia; however, her atherosclerotic cardiovascular disease risk score remains low. She has tolerated the combination therapy well without infectious complications and has received vaccination against herpes zoster. She is ultimately planned to continue dual targeted therapy with a reduced dose of upadacitinib (15 mg)

## DISCUSSION

Dual targeted therapy is an emerging strategy for refractory IBD, allowing inhibition of complementary inflammatory pathways. Although Janus kinase-1 inhibitors provide broad suppression of multiple cytokine signals, they typically spare the IL-23 pathway; conversely, risankizumab offers highly selective blockade of the IL-23 p19 subunit.^4^ Combining these treatments creates a synergistic effect by closing gaps in the inflammatory signaling network.

Prior reports have described combinations of biologics with small-molecule agents or dual biologic therapy with encouraging results.^5–7^ Safety remains a critical consideration with dual targeted immunosuppression therapy, as prior analyses of dual targeted regimens in IBD highlight an elevated infection risk with combination therapy, underscoring the need for careful patient selection and monitoring.^8^

To our knowledge, this case illustrates the first instance of combination therapy with risankizumab and upadacitinib in UC in a patient presenting with a steroid refractory UC flare with rapid and sustained clinical, endoscopic, and histological remission. Apart from mild hypercholesterolemia, no major adverse events were observed for 10 months of follow-up, with serial monitoring of complete blood count/liver profile and lipid profile, suggesting that with close follow-up and risk stratification, dual targeted therapy may be a feasible option for selected patients with refractory UC. However, further studies are needed to define safety, efficacy, and appropriate patient selection for dual targeted therapy in UC.

## DISCLOSURES

Author contributions: P. Srivoleti: study conception, interpretation of data, critically revising the article, final approval of the version to be submitted. N. Dhillon: acquisition of data, drafting the article, final approval of the version to be submitted. L. Boneschansker: study conception, analysis of data, critically revising the article, final approval of the version to be submitted. L. Boneschansker is the article guarantor.

Financial disclosure: L. Boneschansker received research support from AbbVie. N. Dhillon and P. Srivoleti have no financial disclosures to report.

Informed consent was obtained for this case report.

## ABBREVIATIONS:

CRP: C-reactive protein
IBD: inflammatory bowel disease
IL: interleukin
UC: ulcerative colitis
